# Closest string with outliers

**DOI:** 10.1186/1471-2105-12-S1-S55

**Published:** 2011-02-15

**Authors:** Christina Boucher, Bin Ma

**Affiliations:** 1 David R. Cheriton School of Computer Science, University of Waterloo, Waterloo, ON

## Abstract

**Background:**

Given *n* strings *s*_1_, …, *s_n_* each of length ℓ and a nonnegative integer *d*, the CLOSEST STRING problem asks to find a center string *s* such that none of the input strings has Hamming distance greater than *d* from *s*. Finding a common pattern in many – but not necessarily all – input strings is an important task that plays a role in many applications in bioinformatics.

**Results:**

Although the closest string model is robust to the oversampling of strings in the input, it is severely affected by the existence of outliers. We propose a refined model, the CLOSEST STRING WITH OUTLIERS (CSWO) problem, to overcome this limitation. This new model asks for a center string *s* that is within Hamming distance *d* to at least *n – k* of the *n* input strings, where *k* is a parameter describing the maximum number of outliers. A CSWO solution not only provides the center string as a representative for the set of strings but also reveals the outliers of the set.

We provide fixed parameter algorithms for CSWO when *d* and *k* are parameters, for both bounded and unbounded alphabets. We also show that when the alphabet is unbounded the problem is W[1]-hard with respect to *n – k*, ℓ, and *d*.

**Conclusions:**

Our refined model abstractly models finding common patterns in several but not all input strings. We initialize the study of the computability of this model and show that it is sensitive to different parameterizations. Lastly, we conclude by suggesting several open problems which warrant further investigation.

## Background

Finding similar regions in multiple DNA, RNA, or protein sequences plays an important role in many applications, including universal PCR primer design [1-4], genetic probe design [2], antisense drug design [2,5], finding transcription factor binding sites in genomic data [6], determining an unbiased consensus of a protein family [7], and motif-recognition [2,8,9]. The CLOSEST STRING problem formalizes these tasks and can be defined as follows: given a set of *n* strings *S* of length ℓ over the alphabet Σ and parameter *d*, the aim is to determine if there exists a string *s* that has Hamming distance at most *d* from each string in *S*. The optimization version of this problem tries to minimize the parameter *d*. We refer to *s* as the *center string* and let *d*(*x*, *y*) be the Hamming distance between strings *x* and *y*.

The CLOSEST STRING was first introduced and studied in the context bioinformatics by Lanctot *et al*. [*2*]. Frances and Litman [10] showed the problem to be NP-complete even in the special case when the alphabet is binary, implying there is unlikely to be a polynomial-time algorithm for this problem unless P = NP. Since its introduction the investigation of efficient polynomial time approximation algorithms and exact exponential time algorithms for the CLOSEST STRING problem has been thoroughly considered [2,11-16].

The CLOSEST STRING problem requires that the Hamming distance constraint be satisfied for each of the input strings and therefore, is robust to the oversampling of the input strings. For this reason it is frequently used to model many of the aforementioned applications. However, this property also causes a severe problem: if the input includes a string that is significantly different from the other input strings, which we refer to as an “outlier”, then it will have the effect of causing there not to exist a center string for the complete set of input strings; *d* will have to be increased dramatically to account for this string and obtain a center string. This is a significant limitation for applications such as the design of universal primers where a small *d* is crucial for the effectiveness of the primers. In this and many other applications, it would be preferable to determine a “good” center string (*i.e*. one that is reasonably close to each of the strings) for a large portion of the input strings rather than trying to find a center string for the complete set and in doing so finding one that is far distance from many or all of the strings. Hence, we aim to model the task of finding a center string that is within distance *d* to most – but not necessarily all – of the input strings, where *d* is reasonably small. Another compelling consequence of the modification of the model is that in situations where a more satisfying solution can be found by regarding a few strings as outliers, the initial decision of including them requires reexamination.

We formally model this problem as follows:

CLOSEST STRING WITH OUTLIERS (CSWO)

INPUT: A set of *n* length-ℓ strings *S* = {*s*_1_,…, *s_n_*} over a finite alphabet Σ and nonnegative integers *k* and *d*.

QUESTION: Find a center string *s* and a subset of S* ⊂ *S*, such that |S*| = *n – k* and *d*(*s*, *t*) *≤ d* for *t* ∈ S*.

For the rest of the paper we denote *n – k* as n*, and *s_i_* [*p*] to be the symbol at position *p* of string *s_i_*.

There exists a simple reduction from the CLOSEST STRING problem to CSWO that demonstrates it is NP-complete even in the special case where the alphabet is binary and *k* = 0, implying it is unlikely to be solved exactly by a polynomial-time algorithm, unless P=NP. One approach to investigating the computational intractability of CSWO is to consider its parameterized complexity, which aims to classify computationally hard problems according to their inherent difficulty with respect to multiple parameters of the input. If it is solvable by an algorithm that is polynomial in the input size and exponential in parameters that are typically small then it can still be considered tractable in some practical sense.

For unbounded alphabet size, we show that CSWO is W[1]-hard for every combination of the parameters ℓ, *d*, and n* and thus, is fixed parameter intractable when parameterized by any subset of these parameters, unless FPT = W[1]. We also show that when the alphabet is unbounded, there exists a fixed parameter tractable algorithm for CSWO with respect to the parameters *d* and *k*. In the case of constant size alphabet, CSWO is fixed parameter tractable for the parameter *n* but intractable for the parameter *k*. The complexity of the problem remains open when parameterized by *d* and the alphabet is of constant size, and when parameterized by n* and *k*.

### Previous Results

It is worth noting that analogous parameterized complexity studies have been performed for the CLOSEST STRING problem and the CLOSEST SUBSTRING problem. Gramm *et al*. [*13*] demonstrated that the CLOSEST STRING problem is FPT when the number of strings remains fixed. This FPT result is based on an integer linear programming formulation with a constant number of variables (assuming *n* is fixed), and the application of the result of Lenstra [19] that proves integer linear programming is polynomial-time solvable when the number of variables remains fixed. They further demonstrated that the problem is FPT when *d* is a parameter by giving a *O*(*n*ℓ + *nd*(*d* + 1)*^d^*) time algorithm [13]. Ma and Sun gave an *O*(*n*|Σ|*^O^*^(^*^d^*^)^) algorithm, which is a polynomial-time algorithm when *d* = *O*(log *n*) and Σ has constant size [16]. Chen *et al*. [*20*], Wang and Zhu [21], and Zhao and Zhang [22] improved upon the fixed parameter tractable result of Ma and Sun [16].

The CLOSEST SUBSTRING problem seems to be inherently more intractable then the CLOSEST STRING problem. Given *n* strings *s*_1_, *s*_2_,…, *s_n_* over alphabet Σ and integers *d* and ℓ, the CLOSEST SUBSTRING problem aims to determine whether there is a string *s* of length ℓ such that, for all *i* = 1,…, *n*, *d*(*s*, ) ≤ *d* where  is a length ℓ substring of *s_i_*. Fellows *et al*. [11] showed that CLOSEST SUBSTRING is W[1]-hard with respect to the number of input strings *n* even for a binary alphabet. When Σ is unbounded the problem is W[1]-hard with respect to the parameters ℓ, *d* and *n*[11]. Most recently, Marx [23] proved the problem is W[1]-hard with combined parameters *n* and *d* even if the alphabet is binary, which resolved an open problem stated in [11,12,24].

## Methods

We give insight into the computational tractability of CSWO through studying the parameterized complexity of the problem. Parameterized complexity aims to classify problems according to their inherent difficulty with respect to multiple parameters of the input.

### Parameterized Complexity

A problem *φ* is said to be *fixed parameter tractable* with respect to parameter *k* if there exists an algorithm that solves *φ* in *f*(*k*) *· n^O^*^(1)^ time, where *f* is a function of *k* that is independent of *n*[*17*]. Given a graph *G* = (*V*, *E*) with vertex set *V*, edge set *E*, and positive integer *k*, the Vertex Cover problem aims to discern where there is a subset of vertices *V_c_* ⊆ *V* with *k* or fewer vertices such that each edge in *E* has at least one its endpoints in *V_c_* . The vertex cover problem is NP-complete [18] but is fixed parameter tractable since there exists algorithmic solutions that have running time *O*(*kn* + 1.3*^k^*) [17]. The corresponding complexity class is FPT.

Not all NP-complete problems are in FPT. For example, consider the NP-complete CLIQUE problem: given an undirected graph *G* = (*V*, *E*) and a positive integer *t*, the aim is determine whether there is a subset of vertices *C* ⊆ *V* of size at least *t* where each pair of vertices in *C* are connected by an edge. The best known algorithms for solving clique runs in time *O*(*n^o^*^(^*^t^*^)^) and hence, there is no known algorithm for solving *t* for which *t* is not in the exponent of *n* in the running time [17].

In order to characterize those problems that do not seem to admit a fixed parameter efficient algorithm, Downey and Fellows [17] defined a *fixed parameter reduction*. We will restrict interest to the W[1] class and hence, the following definition will only apply to W[1]-hardness. W[1]-hardness gives convincing evidence that a parameterized problem with parameter *k* is unlikely to have an algorithm that has running time of the form *f*(*k*) *· n^O^*^(1)^. Let *L*, *L*′ ⊆ Σ* ×ℕ be two parameterized languages, then *L reduces* to *L*′ if there are functions *k → k*′ and *k → k*″ from ℕ to ℕ and a function (*x*, *k*) *→ x*′ from Σ* *×*ℕ to Σ* such that:

1. (*x*, *k*) *→ x*′ is computable in time *k*″|*x*|*^c^*, for some constant *c* and

2. (*x*, *k*) ∈ *L* if and only if (*x*′, *k*′) ∈ *L*′.

## Results and Discussion

In the following subsections, we study the parameterized tractability of CSWO and show the problem is sensitive to different parameterizations.

### CSWO: Tractability Results

We first consider when Σ is a parameter. In computational biology applications the biological sequences of interest are typically DNA or protein sequences, hence the number of different symbols is a small constant (*i*.*e*. 4 or 20 in the case of DNA or protein sequences, respectively). Restricting Σ only does not make CSWO tractable since it is NP-hard even when the alphabet is binary. However, if Σ and ℓ are both parameters then it is fixed-parameter tractable; we can enumerate and check all the |Σ|^ℓ^ possible center strings. As a result the problem is fixed parameter tractable with the combined parameters Σ, ℓ, *d* and n*. We will prove in a later section that it is imperative that Σ be a parameter in order to obtain this tractability.

Next we show that CSWO is fixed parameter tractable if *d* and *k* are parameters. The fixed parameter algorithm that we present is similar to the algorithm presented by Gramm *et al*. [*13*], where it is proved that CLOSEST STRING is fixed parameter tractable with respect to the parameter *d*. In the algorithm by Gramm *et al*. [*13*] at each recursive step a string *s* is selected that has Hamming distance at least *d* + 1 away from the current candidate center string *x* if one exists; otherwise *x* is returned since it is a center string. Then for any *d* + 1 positions where *x* and *s* disagree, there is at least one position at which *s* is equal to the final solution. The algorithm tries each of the *d* + 1 positions, changes *x* to *s* at one of the *d* + 1 the position, reduces Δ*d* by one, and calls itself recursively. Hence, Δ*d* is the current degeneracy parameter at a particular recursive iteration and *x* is the current candidate center string. Since the recursion stops after at most *d* steps the size of the search tree is bounded by *O*((*d* + 1)*^d^*).

### CSWO Algorithm

**Input:** A CSWO instance with a set of *S n* strings of length ℓ, parameters Δ*d*, *d* and *k*, and a candidate string *x*.

**Output:** A string s* if there exists a set *S* of at least n* strings where each string in *S* has distance at most *d* from s*, and “Not found” otherwise.

1. If Δ*d <* 0 or *k <* 0 then return “Not found”.

2. Choose *i* ∈ {1,…, *n*} such that *d*(*x*, *s_i_*) *> d*. If no such *i* exists return *x*.

3. *s_ret_* = CSWO Algorithm (*S\* {*s_i_*}, Δ*d*, *k –* 1, *x*).

4. If *s_ret_* = “not found ” then:

(a) ***P*** = {*p* | *x*[*p*] ≠ *s_i_*[*p*]};

(b) Choose any ***P***′ from ***P*** with |***P***′| = *d* + 1.

(c) For each position *p* ∈ ***P***′*:*

*•* Let *x* be equal to *s_i_* at position *p*.

*• s_ret_* = CSWO Algorithm (*S*, Δ*d –* 1, *k*, *x*).

*•* If *s_ret_* ≠ “not found”, then return *s_ret_*.

5. Return “not found”.

Our algorithm begins with *s*_1_ as the candidate center string. If *s*_1_ is a center string with respect to *S* then we are done; otherwise there exists a string *s_i_* that has distance at least *d* + 1 from *s*_1_. We “guess” whether *s_i_* belongs in the set of outliers. If it is an outlier then we remove it from *S* and recurse on the smaller set with *k –* 1. If it is not an outlier then we use *s_i_* to move the candidate string *x* closer to toward *s_i_*, which can be done by applying the methodology of Gramm *et al*. [*13*]. We use the term “guess” as an euphemism in this brief description of the our algorithm but rather we try both possibilities as can be seen in the CSWO Algorithm. This will increase the size of the search tree.

**Proposition 1***The CSWO Algorithm solves the CSWO problem in time O*(*n*ℓ *+ nd · d^d^ · 2^k+d^*).

**Proof. Running time.** Each recursion of the algorithm reduces either *k* or *d* by 1. Thus, there are at most *k* + *d* guesses of whether a particular string belongs in the set of outliers. Thus, the search tree size is increased by a multiplicative factor of at most 2*^k^*^+^*^d^* and the search tree size is bounded above by *O*(2*^k^*^+^*^d^ ·* (*d* + 1)*^d^*). The analysis of Gramm *et al*. [*13*] demonstrated that each recursive step takes time *O*(*nd*) and the preprocessing time takes *O*(*n*ℓ) and therefore, we obtain an overall running time of *O*(*n*ℓ + *nd · d^d^ ·* 2*^k^*^+^*^d^*).

**Correctness** We show the correctness of the algorithm by showing the correctness of the first recursive step and then the correctness of the algorithm follows by inductively applying the following argument. Clearly, if *S* does not contain a subset S* of n* strings, such that there exists a center string s* for S* then “not found” will be returned and therefore, we assume otherwise.

If *s*_1_ is a center string for *S* then the algorithm immediately halts so we assume there exists a string *s_i_* in *S* that does not have *s*_1_ as a center string. CSWO Algorithm creates two subcases: one where *s_i_* is in the set of outliers, and another where *s_i_* is not. Suppose *s_i_* is in the set of outliers then the first case will successfully remove *s_i_* from the set and recurse on *S\*{*s_i_*}. Otherwise, if *s_i_* is not in the set of outliers then eventually the second case will reached. We refer to the set of positions as *correct* if {*p* | *s*_1_[*p*] ≠ s*[*p*] = *s*[*p*]}. It follows from Gramm *et al*. [*13*] that one of the *d* + 1 chosen positions *p* will be a correct one. Thus, we have shown that either one of the subcases will lead to a smaller subcase containing the solution for *S*.

The previous result demonstrates the fixed parameter tractability with respect to *d* and *k*. We note that a similar modification of the *O*(*n*|Σ|*^O^*^(^*^d^*^)^) algorithm of Ma and Sun [16] also gives a fixed parameter algorithm with respect to the parameters Σ, *d* and *k*. In the modified algorithm, for any string *s* with distance greater than *d* to the current candidate center string *x*, we again try the subcases where *s* is an outlier, and is not an outlier. In the former case, we remove *s* from the set of input strings *S* and recurse on *S* and *k –* 1, and in the latter case, we use the same technique as in the algorithm of Ma and Sun [16] to reduce the distance between *x* and the final solution. This modification that accounts for the outliers results an extra multiplicative factor of *O*(2*^k^*^+log^*^d^*) to the running time of the original algorithm. Although this algorithm improves upon the running time of the previous result, it requires that Σ is also a parameter. Further, we note that some of the recent improvements [20-22] to the algorithm of Ma and Sun can be modified in a similar manner to obtain fixed parameter algorithms for CSWO with respect to parameters Σ, *d* and *k*.

**Proposition 2** CSWO *is fixed parameter tractable for parameters* Σ *and n*.

**Proof.** Gramm *et al*. [*13*] gave a linear fixed parameter tractable algorithm for CLOSEST STRING with respect to the number of strings and Σ, which we refer to this algorithm as *ILP-procedure(S)*, where *S* is the set of input strings. Our algorithm enumerates all size-n* subsets of *S*, and call *ILP-procedure* on each subset.

### CSWO: Intractability Results

We derive the W[1]-hardness result by a series of intermediate steps, aiming at a reduction from Clique to CSWO, showing that CSWO is W[1]-hard for the combination of ℓ, *d*, and n*, and when the alphabet is unbounded.

#### Reduction from CLIQUE

As previously described, we let the CLIQUE instance be given by an undirected graph *G* = (*V*, *E*) with a set *V* = {*v*_1_,*v*_2_,…,*v_n_*} of *n* vertices, a set *E* of *m* edges, and a positive integer *t* denoting the size of the desired clique. We describe how to generate a set *S* of  strings such that *G* has a clique of size *t* if and only if there is a subset of *S* of size , denoted as S*, where there exists a string *x* such that *d*(*s_i_*,*x*) ≤ *d* for all *s_i_* ∈ S*. We let ℓ = *t* and *d* = *t –* 2. We assume that *t >* 2 since *t* ≤ 1 produces trivial cases.

We begin by describing the alphabet. We assume |Σ| can be infinite and we let Σ be equal to the union of the following sets of symbols:

1. {*v_i_*| for all *i* = 1,…, |*V*|}. Hence, there exists one symbol representing each vertex in *G*.

2. {*c_i_*_,_*_j_*_,_*_m_*|*i* = 1,…,*t*; *j* = 1,…,*t; m* = 1,…, |*E*|}. There exists an unique symbol for each  strings produced for our reduction.

Hence, we have a total of  number of symbols.

Next, we generate a set of  strings *S* = {*s*_1,1,1_,…, *s*_1,1,|_*_E_*_|_, *s*_1,2,1_,…, *s*_1,2,|_*_E_*_|_,… ,*s_t–_*_1,_*_t_*_,|_*_E_*_|_}. Every string has length *t* and will encode one edge of the input graph. There will be  corresponding for each edge, however, encode the edges in different positions. For string *s_i_*_,_*_j_*_,_*_m_* we encode edge *e_m_* = (*v_r_*, *v_s_*), where 1 ≤ *r < s* ≤ |*V*|, but letting position *i* equal to *v_r_* and position *j* equal to *v_s_* and the remaining positions equal to *c_i_*_,_*_j_*_,_*_m_*. Hence, a string is given by

s_i,j,m_ := [c_i,j,m_] ^i–1^ v_r_[c_i,j,m_] ^j–i–1^ v_s_[c_i,j,m_]^m–j^.

**Figure 1:**
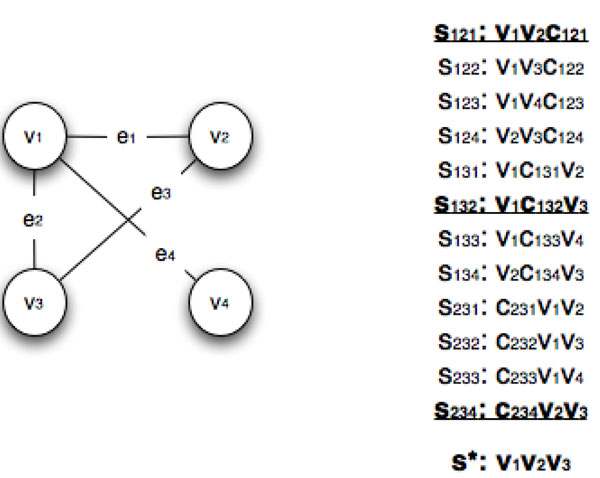
**An illustration of the reduction from CLIQUE to CSWO** Example of the reduction from a CLIQUE instance *G* with *t* = 3 to an instance of CSWO with 12 strings with ℓ = *t* = 3, *d* = *t –* 2 = 1, and . In bold we have the set of strings S* = {*s*_121_, *s*_132_, *s*_234_} that corresponds to the clique containing the vertices {*v*_1_,*v*_2_,*v*_3_}. We note that S* is the only set such that |S*| = 3, which have Hamming distance at most *d* from s*.

To clarify our reduction, we give an example. Let *G* = (*V*, *E*) be an undirected graph with *V* = *v*_1_, *v*_2_, *v*_3_, *v*_4_ and edges *E* = {(*v*_1_, *v*_2_), (*v*_1_, *v*_3_), (*v*_1_, *v*_4_), (*v*_2_, *v*_3_)} and let our CLIQUE instance have *G* and *t* = 3. Figure 1 illustrates the reduction. Using *G*, we exhibit the above construction of  strings, which we denote as *S*. We claim that there exists a clique of size 3 if and only if there exists a string s* of length ℓ = *t* = 3 and subset S* of *S* of size 3 where *d*(*s*,*s_i_*) ≤ *d* for all *s_i_* ∈ S*. In this example the center string *s* is equal to *v*_1_*v*_2_*v*_3_ and each string in the set {*v*_1_*v*_2_*c*_121_, *v*_1_*c*_132_*v*_3_, *c*_234_*v*_2_*v*_3_} is such that each string in S* has Hamming distance at most 1 from *s*.

#### Correctness of the Reduction

The following two lemmas establish the correctness of the reduction.

**Lemma** 1 For a graph with a t-clique, the construction in Subsection produces a CSWO instance with a set S∗ and a string s of length such that for every s_i_ ∈ S* d(s_i_, s) ≤ d.

**Proof** Let the input graph have a clique of size *t*. Let *v_α_*_1_, *v_α_*_2_, …, *v_αt_* be the vertices in the clique *C* of size *t* and without loss of generality, assume *α*_1_*< α*_2_*<…< α_t_* . Then we claim that the there exists a subset of  vertices that have distance at most *t –* 2 from the string *s* = *v_α_*_1_*v_α_*_2_ …*v_αt_*. Consider the first edge of the clique (*v_α_*_1_, *v_α_*_2_) of the clique then it follows that the string *s*_11_*_r_* = *v_α_*_1_*v_α_*_2_ [*c*_11_*_r_* ]*^t–^*^2^, where edge *r* has endpoints *v_α_*_1_*v_α_*_2_, is contained in the set of strings {*s*_111_, *s*_112_,…, *s*_11|_*_E_*_|_}. Clearly, *H*(*s*_11_*_r_*,*s*) = *t –* 2. For each edge in *C* we have we have a string in *S* that has distance at most *t –* 2 from *s* and our lemma follows from this construction.

For the reverse direction, we need to prove that the existence a subset S* of  and a string *s* where *d*(*s*, *s_i_*) ≤ *t –* 2 for all *s_i_* ∈ S* implies the existence of a clique in *G* with *t* vertices.

**Lemma 2***The t symbols of the center string correspond to the t vertices of clique in the input graph*

**Proof.** Let S* be the subset of *S* of size  such that *s* has distance *t –* 2 from each string in S*. Since ℓ = *t*, n* = *t*, *d* = *t –* 2 and for each symbol *c_i_*_,_*_j_*_,_*_m_* there exists only a single string *i* = 1, …,*t*, *j* = 1, …,*t* and *m* = 1,…, |*E*| it follows from the Pigeonhole principle that the center string *s* only contains symbols from {*v_i_*| for all *i* = 1,…,|*V*|}. Without loss of generality assume *s* is equal to *v_α_*_1_*v_α_*_2_ …*v_αt_* for *α_v_*_1_, *α_v_*_2_,…,*α_vt_* ∈ {1,…, |*V*|}. Consider any pair *α_i_*, *α_j_* for 1 ≤ *i < j* ≤ *t* and consider the set of strings *S_i_*_,_*_j_* = {*s_i_*_,_*_j_*_,1_, *s_i_*_,_*_j_*_,2_,…, *s_i_*_,_*_j_*_,|_*_E_*_|_}. Recall that *S_i_*_,_*_j_* contains a string corresponding to each edge *e* = (*r*, *s*) in *E* which has *v_r_* at the *i*th position and *v_s_* at the *jth* position and *c_i_*_,_*_j_*_,_*_m_* at all remaining positions. Therefore, we can only find a string in *S_i_*_,_*_j_* that has distance at most *t –* 2 from *s* if *v_α_í__* is at the *i*th position and *v_αj_* is at the *j*th position; and such a string exists if and only if there is an edge in *G* connecting *v_αí_* to *v_αj_*. Hence, the center string *s* implies there exists an edge between any pair of vertices in *G* in the set {*v_α_*_1_*v_α_*_2_ … *v_αt_*} and by definition the vertices form a clique.

Our main theorem follows directly from Lemma 1 and Lemma 2. We note that the hardness for the combination of all three parameters also implies the hardness for each subset of the three.

**Theorem 1** CSWO *with unbounded alphabet is W[1]-hard with respect to the parameters* ℓ, d, and n*.

Since there exists a trivial reduction from the CLOSEST STRING problem to CSWO (*i*.*e*. simply set *k* = 0 in CSWO), there cannot exist a fixed parameter tractable algorithm for CSWO with *k* as a parameter, unless P = NP; such an algorithm would contradict the NP-hardness of CLOSEST STRING.

**Fact 1** CSWO *is W[1]-hard with respect to the parameter k and when* |Σ| ≤ 2, *unless P = NP*.

## Conclusions

We introduced the CSWO problem, and proved with unbounded alphabet size and parameterized by ℓ, *d* and n* it is W[1]-hard. We also gave fixed parameter algorithms for the problem when parameterized by *d* and *k*, and with unbounded alphabet size. In the case of a fixed alphabet size, we showed CSWO is fixed parameter tractable when parameterized by *n* = n* + *k*. Table 1 summarizes these tractability and intractability results.

Currently, the fixed parameter tractability of the CSWO problem when parameterized by *d*, n* and Σ, and by n* and *k*, remains open (see Table 1). In addition, the existence of efficient, non-trivial approximation algorithms for this problem warrants further investigation.

**Table 1:** Parameterized tractability of CSWO

Parameter(s)	|Σ| is a parameter	|Σ| is unbounded
ℓ, *d*, n*	FPT (trivial)	W[1]-hard (*)
ℓ	FPT (trivial)	W[1]-hard (*)
*d*, n*	Open	W[1]-hard (*)
*d*, *k*	FPT (*)	FPT (*)
n*, *k*	FPT	Open
*k*	W[1]-hard (trivial)	W[1]-hard (trivial)

An overview of the fixed parameter tractability and intractability of the CSWO. Asterisk denotes the new results found in this paper.

## Competing interests

The authors declare that they have no competing interests.

## Authors’ contributions

Concept, FPT analysis: BM. W[1]-hardness analysis: CB. Manuscript preparation: BM and CB.
